# Current status and challenges of immunotherapy in ALK rearranged NSCLC

**DOI:** 10.3389/fonc.2022.1016869

**Published:** 2022-12-14

**Authors:** Rongbin Qi, Yingying Yu, Mo Shen, Dongqing Lv, Susu He

**Affiliations:** ^1^ Department of Respiratory Medicine, TaiZhou Hospital of Zhejiang Province Affiliated to Wenzhou Medical University, Linhai, Zhejiang, China; ^2^ The First Clinical Medical College of Zhejiang Chinese Medical University, Hangzhou, China; ^3^ Department of Respiratory Medicine, At Enze Hospital, Affiliated Taizhou Hospital of Wenzhou Medical University, Taizhou, Zhejiang, China

**Keywords:** ALK, NSCLC, immunotherapy, immune checkpoint inhibitor, process

## Abstract

Rearrangements of the anaplastic lymphoma kinase (ALK) gene account for 5-6% in non-small cell lung cancer (NSCLC). ALK rearranged NSCLC is sensitive to ALK tyrosine kinase inhibitors (TKIs) but prone to drug resistance. Meanwhile, ALK rearranged NSCLC has poor response to single immunotherapy. Here we mainly describe the immune escape mechanisms of ALK mutated NSCLC and the role of related biomarkers. Additionally, we collate and evaluate preclinical and clinical studies of novel immune combination regimens, and describe the prospects and perspectives for the *in vivo* application of novel immune technologies in patients with ALK rearranged NSCLC.

## Introduction

Nearly 80% of lung cancer is NSCLC, one of the leading causes of death due to cancer today (1, 2). Oncogenic drivers of NSCLC including the Echinoderm Microtubule associated protein Like 4-anaplastic lymphoma kinase (EML4-ALK) fusion were first demonstrated by soda et al (3). More than 20 fusion partners of ALK have been identified in NSCLC, with the EML4-ALK fusion being the most common and ALK translocations present in approximately 3% - 5% of patients with non-squamous NSCLC, who are mostly young women who are light or have never smoked (3–5).

**Figure 1:**
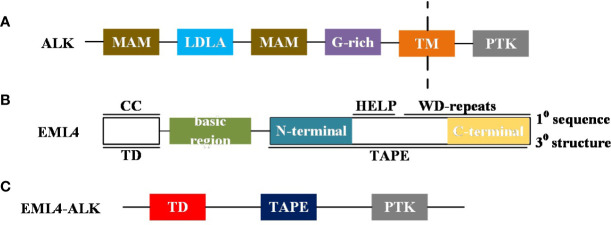
**(A)** A brief diagram of the ALK domain structure: MAM, Meprin, A5 protein and protein tyrosine phosphatase Mu domain; LDLa, Low-density lipoprotein receptor domain class A; G-rich, glycrine-rich region; TM, transmembrane helix; PTK, Receptor tyrosine kinase. **(B)** Summary diagram of the domain structure of human EML4: Primary (1°) sequence features: CC, coiled-coil; basic region; HELP, Hydrophobic motif found in EML proteins; WD repeats, Trp-Asp repeats. Tertiary (3°) structure features: TD, trimerisation domain; TAPE, tandem atypical propeller domain found in EML proteins. The TAPE domain N-terminal region is coloured teal and the C-terminal region is coloured canary yellow. **(C)** Schematic representation of EML4-ALK variant proteins:ALK PTK domain insertion into EML4 protein.

The oncogenic role of ALK was first recognized with the discovery of the nucleophosmin-anaplastic lymphoma kinase (NPM-ALK) fusion in anaplastic large cell non-Hodgkin lymphoma (ALCL) (6). Since then, fusions, mutations, and alternative splicing of ALK have been successively detected in other cancers, such as NSCLC, digestive tract cancer, renal cell carcinoma, breast cancer, thyroid cancer, and ovarian cancer, among others (7–9). The EML4-ALK fusion was first detected in a Japanese patient with lung cancer, and this fusion mutation was mutually exclusive with oncogenic driver mutation types such as KRAS or EGFR (3). In addition, the study did not find the mutation status in 261 other malignant tumor samples tested for EML4-ALK fusion, which also indicated that EML4-ALK was a highly specific mutation of NSCLC, and this also provided a basis for specific therapy for NSCLC patients with ALK mutations (3). The EML4-ALK fusion is formed by a small inversion within the short arm of chromosome 2 that connects a different part of the EML4 gene and a part of the ALK gene, but primarily causes fusion of the 5 ′ end ligand of EML4 and the tyrosine kinase coding region of ALK (**Figure 1C**).

The pathogenesis of lung cancer induced by EML4-ALK has been confirmed by evidence, and the rapid development of lung cancer is mainly caused by overexpression of EML4-ALK in type II alveolar epithelial cells through the Clara cell secretory protein (CCSP) or surfactant protein c (SPC) promoter (10, 11). In addition, Maddalo et al. used CRISPR/Cas9 (clustered regularly spaced short palindrome repeats/CRISPR related protein 9) gene to induce EML4-ALK rearrangement in animal models, which also induced lung cancer (12). Chaperones further induce sustained dimerization of the ALK activation domain, sustained activation of the ALK tyrosine kinase and activation of downstream signaling pathways (examples: phosphoinositide 3-kinase-AKT, JAK-STAT pathways, and RAS mitogen activated protein kinase pathways) (3, 13). Activation of these pathways allows uncontrolled cell growth and proliferation, which is the main oncogenic mechanism in ALK fusion mutated NSCLC.

**Figure 2:**
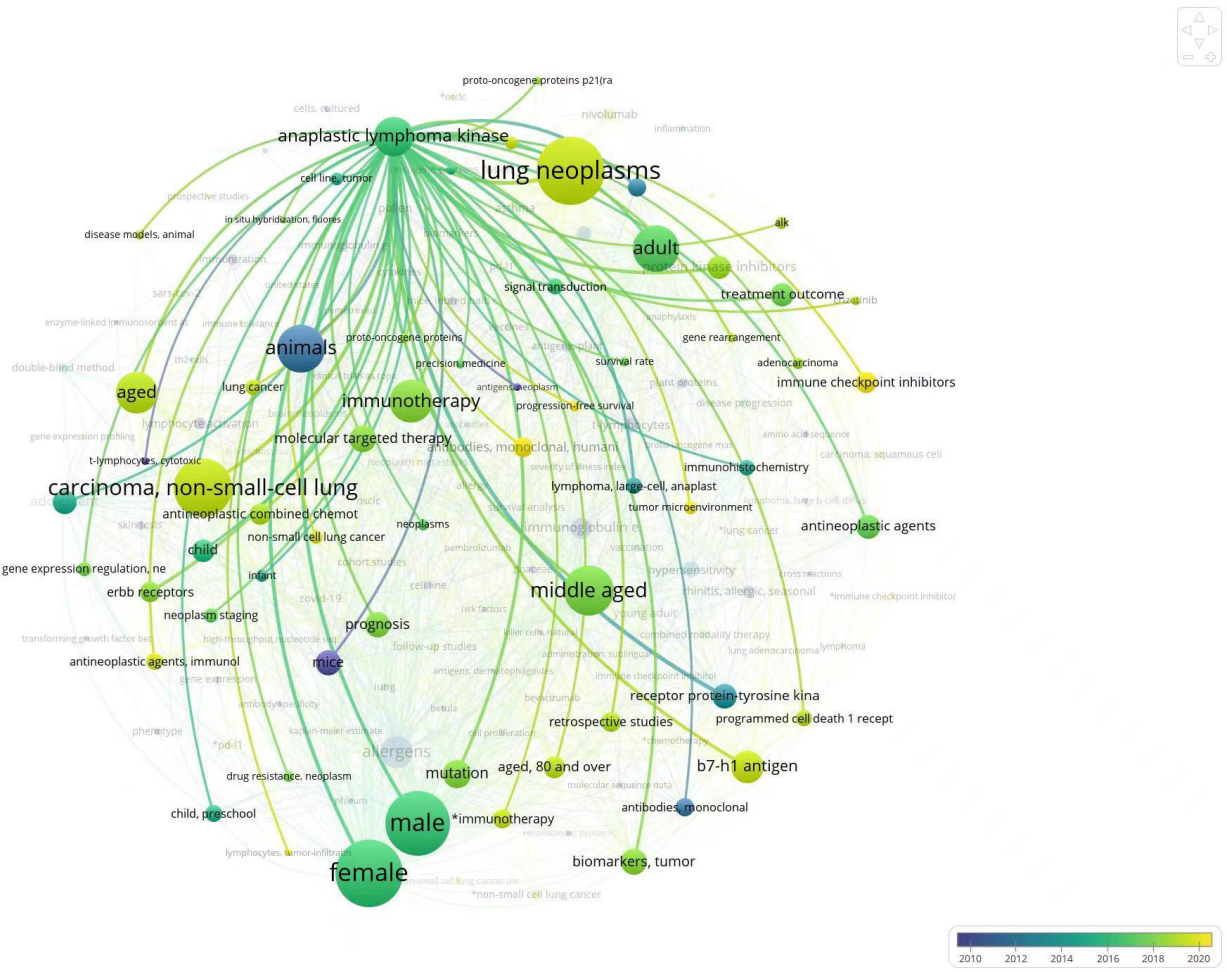
Circles in the figure represent keywords; Circle size represents frequency of occurrence of key words; The connection between the circle and the circle represents the relationship between the keywords, the coarser the connection is; Color represents time.

Anaplastic lymphoma kinase (ALK) tyrosine kinase receptor has been identified as a therapeutic target in lung cancer. We analyzed the direction of recent research on ALK related articles to retrieve ALK related articles through PubMed for the last decade (**Figure 2**). Analysis of the retrieved results found that the main research direction in recent years is ALK mutated NSCLC, at the same time, about the ALK mutated NSCLC of the scheme of immunotherapy and the exploration of related mechanisms. Meanwhile, new advances have emerged regarding the immunotherapy of ALK fusion cancers, such as combination regimens of immunosuppressive agents, tumor vaccines, ALK chimeric antigen receptor (CAR-T) therapy, adoptive T cell therapy, and ALK antibodies. In this review, we discuss immune related parameters associated with oncogenic driver mutations of the ALK rearrangement and outline recent advances in immune related therapies in NSCLC with such mutations.

## ALK related immune mechanisms

Numerous preclinical and clinical studies have suggested that wild-type ALK is predominantly expressed within the peripheral and central nervous system, with little expression in the remaining sites (14). Because the expression of ALK related proteins is largely restricted to the nervous system, making the immune system rarely exposed to ALK protein, it is highly likely that ALK related proteins can be potential antigens. Similarly, cancers driven by ALK fusions or mutations can express specific ALK proteins, which act as neoantigens to elicit an organismal anti-tumor response. The segment of the gene encoding the ALK tyrosine kinase receptor is known, and studies have found that about 80% of its domains are homologous to the leukocyte tyrosine kinase (LTK: a proto oncogene involved in immune related diseases). The human ALK family, like the LTK family, can be activated by the small peptide ligand Family with sequence similarity 150, member A (FAM150A) (Aug β) and Family with sequence similarity 150, member B (FAM150B) (Aug α) Activation (15, 16). The immune related physiological role of ALK is not fully understood, therefore, we collated the studies on the immune related mechanisms of ALK in recent years and summarize them to help gain a more complete understanding of the scientific frontier movement of ALK related immune research, and to analyze the inadequacies of the existing studies.

### Aberrant inheritance of ALK wild type in human cancers

The intracellular kinase domain of ALK is a 220 kDa cell surface tyrosine kinase receptor that belongs to the insulin receptor superfamily. In addition, the ALK extracellular domain includes a glycine rich region, a low-density lipoprotein class a domain, and two MAM regions (meprin/A5 protein/ptpmu) (17, 18) (**Figure 1A**). It is well known that ALK gene rearrangement (fusion), mutation, amplification and alternative splicing events can lead to a variety of cancers, and ALK gene rearrangement is the most common type in various types of cancer. For example, predominant NPM-ALK fusion in ALCL, predominant TPM3/4-ALK fusion in IMT and predominant ALK fusion types such as EML4-ALK fusion in NSCLC (19–21).

**Figure 3:**
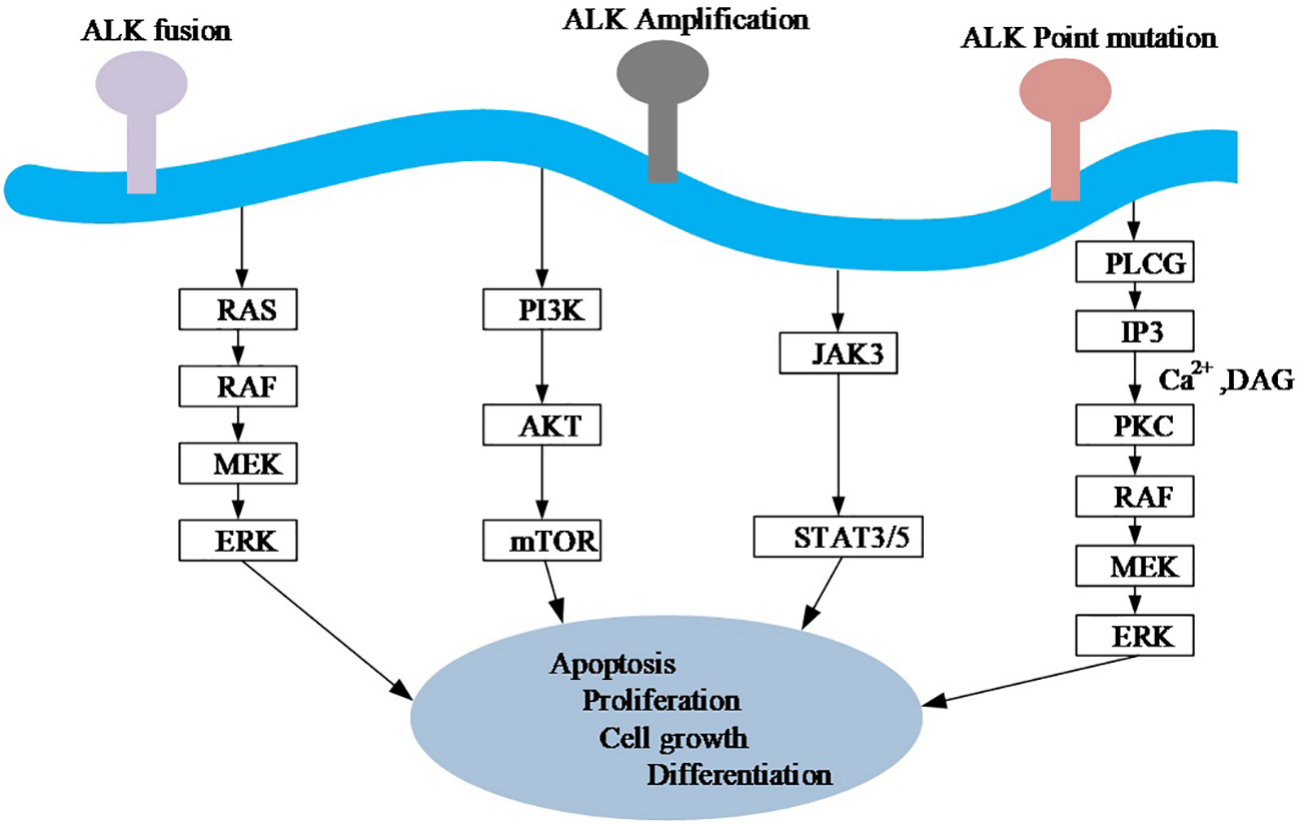
A simplified diagram of oncogenic signaling mechanisms of the ALK gene. Ras, rat sarcoma; Raf, rapidly accelerated fibrosarcoma; MEK, mitogen-activated protein kinase; ERK, extracellular signal-regulated kinase; PI3K, Phosphoinositide 3-kinase; AKT, protein kinase B. mTOR, mechanistic target of rapamycin; JAK, Janus Kinase; STAT, Signal transducer and activator of transcription; PLC G, G protein-phospholipase C. IP3, Inositol triphosphates; PKC, Protein kinase C; DAG, Diacylglycerol.

At the primary sequence level, EML proteins are composed of a number of WD (Trp-Asp) repeats N-terminal to them and a help (hydrophobic Echinoderm Microtubule-Associated Protein (EMAP) like protein) motif present at the N-terminus, whereas at the tertiary sequence level, EML proteins consist of a trimerization domain (TD), a basic region, and a Tandem Atypical Propeller EML (TAPE) domain (**Figure 1B**). ALK mutations have point mutations, gene amplifications and splice isoforms, among others, in addition to fusions. The incidence of ALK point mutations is low, and their oncogenic role has been found only in neuroblastoma (22). The role of the splice isoforms of ALK in tumorigenesis remains undetermined. The current study found that ALK can promote tumor cell proliferation and survival by activating signaling pathways including PI3K, JAK/STAT, and MAPK (**Figure 3**).

### ALK involved innate immunity

There are studies showing the role of ALK in regulating innate immunity during lethal sepsis (23, 24). Mechanistically primarily ALK directly interacts with epidermal growth factor receptor (EGFR), triggering the serine threonine protein kinase AKT to phosphorylate and activate the interferon regulatory factor 3 (IRF3) and Nuclear factor- Kappa B (NF-kB) signaling pathways, enabling a stimulator of interferon genes (STING) dependent stringent inflammatory response. This finding sheds light on the key role of ALK in inflammatory signaling pathways and also on the possible progression of ALK associated tumors by immunotherapy.

### Anti ALK antibody responses against ALK wild type and ALK fusion

Antibodies against the NPM-ALK fusion were first discovered *in vivo* in ALCL patients in the early 2000s by Pulford et al., while antibodies against ALK-WT were also found to be present in 91% of patients (25). A recent study found that anti ALK antibodies were detected within the serum of 21 ALK positive NSCLC patients within the study, but not in healthy adults (26). These data suggest that for ALK positive NSCLC patients, enhancing their pre-existing anti ALK immune response *in vivo* may be more beneficial than for ALK wild-type patients. Meanwhile these findings also suggest that humoral immunity against ALK related proteins may only occur in cancer patients, which will provide an opportunity for immunotherapy of ALK related tumors.

Anti ALK antibodies against ALK-WT and ALK fusions can specifically attack ALK positive tumor cells, contributing to the inhibition of tumor growth. A recent retrospective analysis of ALK autoantibody concentrations in patients with ALCL found that patients with high titer of anti ALK autoantibodies had longer survival and that ALK autoantibody levels inversely correlated with tumor recurrence (27). But less data is available for ALK rearranged NSCLC patients, and more preclinical and clinical studies on the related mechanisms are needed to provide a theoretical basis for relevant immunotherapy regimens in the future.

### Cytotoxic T cell response to ALK

In addition to triggering humoral responses, tumor specific antigens can potentially induce cytotoxic T cell (CTL) responses to eliminate tumor cells. Relevant studies have demonstrated the existence of a precursor pool of anti ALK CD8+T lymphocytes in cancer patients (28). Some studies have found high levels of anti ALK CD8 + T cells in both healthy populations and ALK positive ALCL patients. However, CD8 + T cells in patients with ALK positive ALCL are mainly effector T cells and central memory T cells, whereas they are mainly naive T cells in the healthy population. Interestingly, further studies found that anti ALK CTLs could effectively inhibit the growth of ALK positive lymphoma cells. From the above findings, anti ALK memory T cells derived from cancer patients were found to significantly induce the development of secondary immune responses in the body upon re stimulation with ALK related proteins. Yoshimura et al. identified a novel epitope polypeptide from EML4-ALK fused NSCLC, and at the same time successfully induced a restricted polypeptide specific CTL clone with significant cytotoxic effects on EML4-ALK positive NSCLC cells. This is a vaccine treatment design based on specific CTLs against EML4-ALK positive cancer cells, and further research and analysis are still required in order to realize the application of this type of vaccine in ALK positive NSCLC patients.

### Immune escape of ALK

ALK fusion genes not only cause body CTL response and humoral immunity, but also induce the expression of PD-L1 on tumor cells (29–31). The induction of high expression of programmed cell death-Ligand 1(PD-L1) on the surface of tumor cells by ALK fusion genes may alter the tumor microenvironment, leading to the occurrence of tumor cell immune escape. Relevant preclinical studies have found that ALK fusion associated proteins induce PD-L1 expression mainly through the activation of Hippo, MAPK, and PI3K and other pathways (30, 32). Although upregulation of PD-L1 is a major immune escape mechanism in ALK related cancers, recently, studies have found that ALK related proteins have some regulatory effect on the antigen-presenting human leukocyte antigen (HLA) system (33). ALK inhibitors may thus serve as potential adjuvants for immunotherapy, and studies to enhance the efficacy of immunotherapy by upregulating HLA expression require further exploration.

## ALK and biomarkers for immunotherapy

### PD-L1

**Figure 4:**
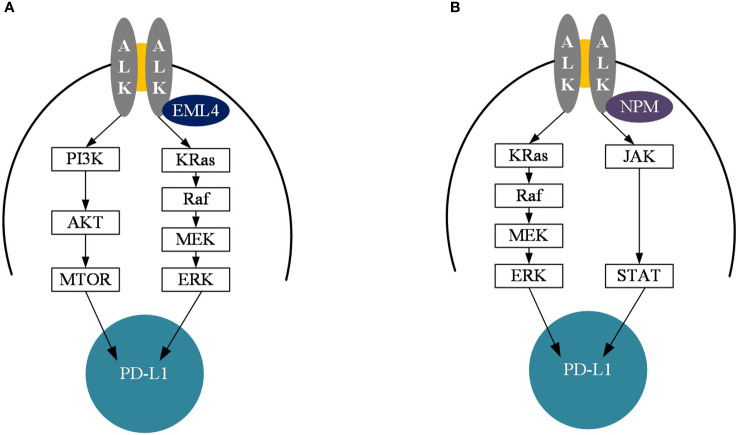
Diagram of the mechanism of PD-L1 upregulation of EML4-ALK and NPM-ALK. **(A)** Diagram of the mechanism of PD-L1 upregulation in EML4-ALK mutant cancers. **(B)** Diagram of the mechanism of PD-L1 upregulation in NPM-ALK mutant cancers. PI3K, Phosphoinositide 3-kinase: AKT, protein kinase; MTOR, mechanistic target of rapamycin; Kras, Kirsten rat sarcoma; Raf, Rapidly accelerated fibrosarcoma; MEK, mitogen-activated protein kinase; ERK, extracellular signal-regulated kinase; JAK, Janus Kinase; STAT, Signal transducer and activator of transcription; ALK, anaplastic lymphoma kinase; PD-L1, Programmed cell death 1 ligand 1.

High expression of PD-L1 and PD-L2 on the surface of tumor cells triggers immune evasion (34, 35). Studies have shown that tumor cells from mice expressing PD-L1 suppress T cell-mediated antitumor immune responses mainly through the PD-1/PD-L1 pathway (36, 37). The effect of EML4-ALK fusion on PD-L1 expression is explained by the findings of Shaodong Hong et al (31). Overexpression of EML4-ALK induced the expression of PD-L1, regulating PD-L1 mainly through p-ERK1/2 and p-AKT, but not through p-JAK3 signaling (**Figure 4A**). Meanwhile PD-L1 upregulation mediated by EML4-ALK fusion protein could induce T cell apoptosis through PD-L1/PD-1 axis. Additionally, survival of EML4-ALK fusion NSCLC was inhibited by anti-PD-1 or ALK inhibitor alone, but synergistic tumor killing effect of ALK inhibitor PD-1 inhibitor was not shown. Another mechanistic study of PD-L1 expression in NPM-ALK fusion positive anaplastic large cell lymphoma found that PD-L1 expression was induced mainly through the MEK-ERK and STAT3 signaling pathways (**Figure 4B**), suggesting that the expression of PD-L1 in ALK fusion is regulated in different ways depending on tumor cell types or differences in ALK fusion partners (38, 39).

Although many findings have shown that the PD-L1 expression rate of EML4-ALK fusion in NSCLC is higher than that of wild type, the poor efficacy of immunotherapy in NSCLC with EML4-ALK fusion is conflicting, and the reason to explain this phenomenon may be the significant difference in study populations and study methods. For example, PD-L1 expression is more variable in ALK fused NSCLC (40, 41), which may be related to sample type, patient population, PD-L1 detection methods, and so on. Additionally, this marker, which poses a major challenge in considering high PD-L1 expression as a biomarker for immunotherapy, is not yet sufficient to completely replace the tumor inflammatory microenvironment. The ability of CD8 positive cytotoxic T lymphocytes to infiltrate the tumor microenvironment determines the sensitivity of NSCLC to immunotherapy, which likewise induces an increase in PD-L1 expression and γ Interferon production (42). However, in the absence of corresponding infiltrating immune cells within the tumour microenvironment, upregulation of PD-L1 is largely achieved through constitutive oncogenic signalling pathways. In fact, one study found that high infiltration of lymphocytes and high expression of PD-L1 rarely co occur in NSCLC patients with EGFR mutations and ALK positive (40). Therefore, such tumors are less responsive to immunotherapy.

### TMB

Tumor mutational burden (TMB) was defined as the total number of detected, somatic gene coding errors, base substitution, gene insertion or deletion errors per million bases, excluding germline mutations (43). Therefore, high and low TMB can suggest genomic stability. The higher the TMB, the higher the gene mutation rate, the more neoantigen production, the more immunogenic the body, and the better the efficacy of immunotherapy (44). Several studies have demonstrated that NSCLC patients with higher TMB have significantly higher clinical benefit from PD-1/PD-L1 inhibitors (45, 46). Findings in a study of 100 patients from Japan (47): Ten NSCLCs had high TMB (> 20 mutations/MB), including two with driver mutations (one ALK rearrangement and one HER2 mutation), whereas 57 of 90 patients’ samples with low TMB had actionable oncogenic driver mutations (ALK, ROS1 or RET rearrangements or EGFR, HER2 or MET mutations) (P < 0.05), suggesting that NSCLCs with oncogenic driver mutations such as ALK rearrangements exhibit low TMB expression. In addition, cancers attributed to carcinogenic exposures (e.g., tobacco, UV), such as NSCLC and melanoma, have generally been found to have higher TMB (48). However, ALK rearrangements were mainly enriched in nonsmoking or smoking limited patients (49, 50), which was consistent with the aforementioned low TMB expression in ALK rearranged lung cancer, which led to the possibility that such tumours might have fewer neoantigens capable of generating immune responses, which might be one of the reasons affecting the efficacy of immunotherapy in patients with ALK rearranged NSCLC.

### Major histocompatibility complex proteins

Major histocompatibility complexes I and II (MHC-I and MHC-II) are key players in the activation and antigen presentation of immune cells of the body. MHC-I is encoded by the HLA-A, - B, and - C genes, and it can be interferon-gamma (IFN-g) and inflammatory pathways such as NF-kB induce expression. Several reasons have been attributed to downregulation of MHC-I expression by cancer cells, including loss of B2M, mutation or deletion of HLA genes and defective trafficking to the cell surface (51). Loss of heterozygosity of HLA genes has been reported in approximately 40% of NSCLC. Meanwhile, loss of heterozygosity in human leukocyte antigen (HLA LOH) has been detected in early lung cancer as a subclonal event, and the gene may be highly expressed in advanced/metastatic tumors, suggesting that this gene may enhance tumor fitness during cancer progression (52). MHC-II is mainly expressed on antigen-presenting cells (e.g., dendritic cells, macrophages, and B lymphocytes, etc.), whereas MHC-II (tumor specific MHC-II) on the surface of tumor cells is mainly IFN-gamma Pathway induced production (53, 54).

T cell immunotherapy is often hampered by limited presentation of tumor specific antigens due to downregulation of human leukocyte antigens (HLA). A recent study found that ALK inhibitors can increase the dosage of HLA associated proteins on the surface of ALK mutated lung cancer cells in both *in vitro* and *in vivo* experiments, while they can decrease PD-L1 levels on the surface of tumor cells by 75%. This suggests that ALK mutant NSCLC may facilitate cancer initiation by enabling tumor evasion of the immune system through downregulation of HLA expression (33). Therefore, the major histocompatibility complex might serve as a biomarker for whether patients with ALK mutant NSCLC can benefit from the combination of ALK inhibitors and T-cell immunotherapy.

### Other biomarkers

Predictive biomarkers of current ICIs treatment efficacy besides PD-L1 expression level, major histocompatibility complexes and TMB, but also TME, microsatellite instability high (MSI-H) and mismatch repair deficiency (dMMR), among others (55–57).

The tumour microenvironment (TME) in ALK rearranged cancers refers to the interplay of CD4 + T cells, CD8 + T cells, tumour associated macrophages (TAMs), mast cells and regulatory T cells (FOXP3 +) (58). Preclinical findings in ALCL suggest that the degree of PD-L1 expression on the surface of tumour cells is positively correlated with TAMs in ALK positive ALCL (59, 60). Furthermore, PD-L1 expressed on the surface of tumor cells and Foxp3 + T regulatory cell interactions lead to diminished CD8 + effector T cell-mediated antitumor responses and cause exhaustion of effector T cells in the body (61). Finally, there are studies that have found that the ratio of CD8 +: Foxp3 + TILs provides more favorable information than either indicator alone, mainly on the reciprocal relationship between responding effector CD8 + TILs and suppressor Foxp3 + T regulatory cells (62).

Recently, studies have also pointed out that mismatch repair (MMR) deficiency and microsatellite instability (MSI) could also predict the therapeutic efficacy of tumor immunotherapy to some extent (63). Defective MMR (dMMR) is a major cause of MSI, so we can indirectly assess whether the function of MMR is precise by detecting MSI. Therefore, MMR and MSI, like TMB, are important indicators for assessing the genomic stability of an organism. Additionally, dMMR induces the synthesis of immunogenic neoantigens by tumour cells, which elicits an immune response in the body against tumour cells, ultimately leading to increased efficacy of immunotherapy in patients with high dMMR (64).

## Methods for detection of ALK alterations

The incidence of ALK rearrangement in lung adenocarcinoma (LADC) is higher than that in lung squamous-cell carcinoma (LSCC) (65, 66). There are four ALK gene detection methods that are widely used in clinical practice now: Ventana D5F3 ALK immunohistochemistry (IHC), fluorescence *in-situ* hybridization (FISH), reverse transcription polymerase chain reaction (RT-PCR), next-generation sequencing (NGS). Fish testing has high sensitivity and specificity to ALK fusion gene detection, and is the gold standard for ALK rearrangement detection (21). Ventana ALK IHC testing is another routine diagnostic method for diagnosing ALK status. It has both excellent sensitivity (100%) and specificity (about 98%). Besides, these two methods have been approved by FDA and NMPA (67). Therefore, Ventana D5F3 ALK IHC method can be used as a promising alternative method for FISH detection or a routine screening method for ALK gene abnormalities in LADC patients (68, 69). There are few studies on ALK fusion gene in lung squamous-cell carcinoma (LSCC) patients. Wang et al. showed that the consistency rate of positive ALK rearrangement detected by IHC method and FISH testing in LSCC patients is extremely low. Therefore, Ventana D5F3 ALK IHC method is not necessarily reliable on the detection of ALK protein level in LSCC patients. So further confirmation by FISH testing or other methods is required (70). With the continuous emergence of tumor biomarkers, the practicability of single gene evaluation has gradually declined. Relevant studies show that NGS method plays an incomparable role in detecting the comprehensiveness of ALK fusion genes and guiding the subsequent therapy after drug resistance. However, the cost of NGS detection method is quite expensive, so it is currently recommended only for NSCLC patients after ALK-TKI resistance (71, 72). RT-PCR method for ALK gene detection is still controversial now. So it is not a regular method for ALK fusion gene detection (73).

## Treatment status

The fusion of EML4-ALK is a unique oncogenic driver mutation in NSCLC, which occurs in approximately 4-8.1% of NSCLC patients (74, 75). Although chemotherapy is still the mainstream treatment for advanced NSCLC with EML4-ALK fusion, ALK tyrosine kinase inhibitors (TKI) (such as crizotinib, brigatinib, alectinib, lorlatinib and ceritinib) have been recommended by FDA for the first-line treatment of NSCLC with advanced EML4-ALK fusion (76). In the PROFILE 1014 trial, the efficacy of crizotinib as first-line treatment for patients with ALK positive advanced non-squamous non-small-cell lung cancer was superior to that of platinum/pemetrexed chemotherapy. Progression free survival (PFS) assessed by radiographic review was statistically significantly longer with chemotherapy (10.9m vs 7.0m P < 0.001) (77). Further evaluating overall survival (OS) in both arms, median OS was not reached with crizotinib (95% CI, 45.8 months to NR) and 47.5 months with chemotherapy (95% CI, 32.2 months to RR) (78). The efficacy and safety of another ALK inhibitor, ceritinib, are similarly significantly superior to chemotherapy regimens (79). Second generation ALK inhibitors (brigatinib, alectinib) have shown efficacy and safety profiles superior to those of crizotinib (80, 81). Additional studies with the third-generation ALK inhibitor lorlatinib found that first-line lorlatinib improved PFS outcomes and reduced CNS progression in patients with advanced ALK positive NSCLC with or without brain metastases at baseline (82). However, most advanced NSCLC patients with EML4-ALK fusion will eventually become resistant to TKIs, and effective means to prevent TKI resistance and further treat TKI resistant patients are currently lacking (83–86). Therefore, new and more effective treatment options are urgently needed for EML4-ALK fused NSCLC. Currently, immunotherapy regimens for a large number of cancers are established after intensive research on immunotherapy (87, 88). In the treatment of patients with NSCLC, the benefit of PD-1/PD-L1 inhibitors is greater compared with that of conventional chemotherapy (89, 90). Studies have shown that PD-L1 expression is upregulated in EGFR mutated and ALK rearranged NSCLC (30, 31, 38, 91), which provides a new idea for clinical treatment strategies for NSCLC patients with EML4-ALK fusion, such as treatment with ICIs alone or in combination with TKI targeted inhibitors. Therefore, we aim to provide direction guidance for future new treatment options for EML4-ALK fusion NSCLC by discussing clinical data on immunotherapy regimens for ALK rearranged cancers.

### ICI monotherapy

Because ALK rearrangements are relatively uncommon in patients with NSCLC, clinical data on single agent ICI therapy for ALK positive NSCLC are mainly derived from retrospective studies and case reports (**Table 1**). Showed in a retrospective study that the objective response rate was 3.6% in ALK positive patients compared with 23.3% in ALK negative patients (40). No significant difference in progression-free survival (PFS) was observed between NSCLC patients with concurrent EGFR mutation/ALK positive and EGFR WT/ALK negative tumors (40). In another retrospective study, evaluation of imaging and survival in 23 patients with ALK rearranged NSCLC found no complete response by imaging in 1 patient, and further survival analysis in the ALK subgroup found Overall Survival (OS) and PFS of 17.0m [3.6; NR], 2.5m [1.5; 3.7], respectively (92). Moreover, Jahanzeb et al. found a similar objective response rate in an efficacy analysis of 83 patients with ALK rearranged NSCLC receiving ICIs, in which the median PFS reached only 3.9 months (93). But in one case report (95), a case of ALK rearranged NSCLC in a 48 year old man was reported. This patient showed a complete response for 16 months under nivolumab therapy in the third line setting after ceritinib and platinum-based chemotherapy.

**Table 1:** Generalizing clinical studies of single immune checkpoint inhibitors in patients with ALK mutated advanced non-small cell lung cancer.

Author/Year	Total No. of Patients	No. of Patients with ALK Rearrangements	Treatment	Endpoint	subgroup		lines of treatment
Justin F. Gainor 2016 (40)	58	6(10.34%)	PD-1/PD-L1 Inhibitors		EGFR mutations or ALK rearrangements	EGFR WT/ALK-negative	Second
				ORR	3.60%	23.30%	
				mPFS	2.07	2.58	
J. Mazieres 2019 (92)	551	23(4.17%)	ICIs		ALK-positive	All cohort	Promiscuous
				DCR	32%	38.29%	
				mOS	17.0m	13.3m	
				mPFS	2.5m	2.8m	
M. Jahanzeb 2021 (93)	83	83(100%)	ICIs		no prior ALK	with prior ALK	Promiscuous
				rwPFS	3.9m	1.5m	
P. Song 2020 (94)	44	3(6.82%)	PD-1 inhibitors.		ALK-positive		
				DCB	100%		Unknow

There are mainly several following prospective randomized clinical trials of single agent ICIs in patients with ALK rearranged NSCLC. In the ATLANTIC study (96), the efficacy and tolerability of durvalumab were primarily assessed in patients who had received at least 2 prior therapies. Interestingly, this study’s subgroup analysis of EGFR mutated and ALK rearranged patients found that objective responses could be observed in NSCLC patients in the EGFR mutated subgroup, but not in the ALK rearranged subgroup, and thus tumors driven by different oncogenes might have different immunobiological effects in response to immune checkpoint inhibitors. However, in another prospective study (94), all NSCLC patients with EML4-ALK rearrangement were found to exhibit durable clinical benefit (DCB) (P = 0.049), suggesting that this gene may be a potential gene for effective immunotherapy. Despite these conflicting observations, further studies with our individual specificity for immunotherapy in this group of patients are needed to better understand the biological mechanisms underlying their response.

To further improve the survival of patients with NSCLC, studies of combination regimens targeting IC have been successively conducted. The results of a recent study found that the combination of nivolumab and ipilimumab is an effective treatment for patients with metastatic NSCLC (97). Although this study excluded patients with ALK rearranged NSCLC, it also provided a new line of research thinking for such patients.

### ICIs + TKI

There are existing clinical studies on the combination therapy of ALK TKI with ICI, and preliminary data suggest good efficacy (**Table 2**). However, the results of various studies are not completely consistent, and drug toxicity induced by simultaneous application cannot be ignored.

**Table 2:** Concluding remarks clinical studies in ALK mutated advanced non-small cell lung cancer receiving immunotherapy combined with targeted therapy.

Study	Total No.of Patients	No. of Patients with ALK Rearrangements	Treatment	Endpoint	subgroup		lines of treatment
D. R. Spigel 2018 (98)	13	13(100%)	nivolumab plus crizotinib		ALK-positive		First
				ORR	38%		
Dong-Wan Kim 2018 (99)	21	21(100%)	alectinib plus atezolizumab		ALK-positive		First
				ORR	81%		
				m PFS	21.7m		
E. Felip 2020 (100)	36	36(100%)	nivolumab plus Ceritinib		450mg	300mg	First/Second
				ORR	83%	60%	
JAVELIN 101	40	28(70%)	Avelumab plus Lorlatinib or Avelumab plus Crizotinib		ALK-positive	ALK-negtive	First
				ORR	46.40%	16.70%	
A. W. Chalmers 2019 (101)	14	3(21.4%)	ipilimumab plus erlotinib or crizotinib		ALK-positive	EGFR-positive	First
				m PFS	24.1m	17.9m	
S. P. Patel 2020 (102)	9	9(100%)	Crizotinib plus Pembrolizumab		dose level 0	dose level -1	First
				ORR	50%	57.20%	
J. J. Lin 2019 (103)	453	345(76.16%)	Crizotinib(plus ICI)		Crizotinib plus ICI	Crizotinib	Promiscuous

Checkmate 370 is a clinical trial for previously untreated advanced ALK rearranged NSCLC patients treated with nivolumab in combination with crizotinib. Of the 13 patients enrolled, 5 (38%) had a partial response, however patient enrollment was stopped due to severe drug toxicity episodes (98). In addition, there are studies that found an acceptable drug safety profile for alectinib combined with atezolizumab antineoplastic therapy, with the major toxic effect being rash, which has a reported incidence of only 18.9% (99). The combination of nivolumab at 3 mg/kg biweekly with either 450 mg/day or 300 mg/day of Ceritinib was evaluated in another phase IB dose escalation trial of 36 patients with ALK rearranged NSCLC. In TKI naive patients with ALK rearranged NSCLC, the ORR was 83% in the population receiving ceritinib 450 mg/day compared with 60% in the population receiving ceritinib 300 mg/day, with an additional finding of higher response rates in the PD-L1 positive subgroup. However, grade 3 rash was reported in 29% and 14% of patients in the 450 mg/day cohort and the 300 mg/day cohort, respectively, none of whom were found to have grade 4 rash (100). The JAVELIN 101 trial evaluated Avelumab plus Lorlatinib or Avelumab plus Crizotinib in patients with ALK rearranged NSCLC. The preliminary data showed that the ORR of Avelumab plus Crizotinib in the treatment of ALK negative patients was 16.7%, but the ORR of Avelumab plus Lorlatinib in the treatment of ALK positive patients was 46.7%, and the safety of Avelumab plus Lorlatinib treatment group was good (NCT02584634) (104). In a phase I trial (101), the CTLA4 inhibitor ipilimumab was evaluated in combination with erlotinib or crizotinib in patients with NSCLC with EGFR mutations or ALK rearrangements. Preliminary data indicated that among three patients with ALK rearranged NSCLC, one patient experienced grade 2 pneumonitis and one patient experienced hypophysitis and discontinued this regimen treatment with a median PFS of 24.1 months.

Recently, Patel et al. analyzed the results of a phase IB study of a combination regimen of pembrolizumab plus crizotinib in patients with previously untreated ALK positive NSCLC. The study terminated the trial early because of dose limiting toxicity occurring in four patients (44%), including hepatotoxicity in three patients (102). In a retrospective analysis (103), the potential toxicity of sequential treatment with PD - (L) 1 inhibitors and crizotinib was highlighted by Lin et al. Among 11 patients who received crizotinib after PD-1 blockade, 5 patients (45.5%) developed grade ≥ 3 hepatotoxicity. In contrast, the incidence of grade ≥ 3 hepatotoxicity was only 8.1% in patients receiving crizotinib alone in this population. From the above findings, it is clear that there is a need for ALK reconstitution before ICI treatment in patients with advanced NSCLC, while drug toxicity studies targeting TKI combined with ICI regimens need to be further explored.

### ICIs combined chemotherapy

As immunotherapy advances, more research teams have pursued the use of ICIs in standard chemotherapy regimens, expecting that more cancer patients will benefit from immunotherapy. Combining immunotherapy with chemotherapy may increase immune system activity through the immune effects of chemotherapy (105), which, for example, causes a decrease in regulatory T cells (106) and diminished activity of myeloid derived cells (107) but increases cross presentation of tumor antigens (108) and induction of PD-L1 expression on the tumor cell surface (109).

But the effect of immunotherapy combined with chemotherapy for ALK rearranged NSCLS remains undefined. Preliminary study data on this treatment option are already available: IMpower150 and IMpower130 (**Table 3**). IMpower150 evaluated atezolizumab in combination with carboplatin/paclitaxel/bevacizumab in patients with NSCLC and found that this combination regimen did not show significant benefit in patients with ALK rearranged NSCLC (112, 113). Similar results were obtained in the IMPOWER 130 study in patients with ALK positive NSCLC who received the addition of atezolizumab to carboplatin and albumin bound paclitaxel without significant benefit (110). A phase II trial recently presented at the World Conference on lung cancer evaluated pembrolizumab in combination with carboplatin and pemetrexed in patients with recurrent EGFR/ALK positive NSCLC previously treated with targeted therapy. Of the 33 patients enrolled, 26 had NSCLCs with EGFR mutations and 7 had ALK fusion positive NSCLCs. In patients with ALK positive tumors, the ORR was 28.6%, while neither mPFs nor mOS showed significant benefit (111). Therefore, the specific efficacy of PD - (L) 1 inhibitor chemotherapy combination for NSCLC patients with ALK recombination has not been clarified, and whether this treatment regimen is beneficial for patients needs further exploration.

**Table 3:** Concluding remarks clinical studies in ALK mutated advanced non-small cell lung cancer receiving immunotherapy combined with chemotherapy.

Study	Total No.of Patients	No. of Patients with ALK Rearrangements	Treatment	Endpoint	subgroup		lines of treatment
H. West 2019 (110)	723		atezolizumab plus chemotherapy		atezolizumab plus chemotherapy	chemotherapy	First
			(carboplatin plus nab-paclitaxel)	m PFS	7.0m	5.5m	
				m OS	18.5m	19.2m	
S. Gadgeel 2021 (111)	33	7(21.21%)	pembrolizumab plus chemotheray		ALK+		Second
				m PFS	2.9m		
				m OS	2.9m		
				ORR	28.60%		

### ICIs combination anti-angiogenic agents

Anti-angiogenic agents induce tumor immune escape, and as immune checkpoint inhibitors are applied to patients with advanced NSCLC, antiangiogenic agents combined with immunotherapy regimens may have synergistic antitumor activity. After ALK TKI resistance, the combination of immunotherapy and anti-angiogenic agents may have greater clinical benefit than chemotherapy alone or in combination with anti-angiogenic agents. Preliminary data from several clinical trials exploring anti-angiogenic agents in combination with immune checkpoint inhibitors have been published (**Table 4**). In the IMpower150 trial (112), the addition of Atezolizumab to bevacizumab, carboplatin, and paclitaxel led to improved median PFS (8.3 months vs. 6.8 months) and median OS (19.2 months vs. 14.7 months) in patients with EGFR or ALK genomic alterations with disease progression on TKIs or who were unable to tolerate TKIs, providing evidence hosting the possibility of application of this regimen in patients with ALK rearranged NSCLC. Similarly, an ongoing multicenter phase II open label nonrandomized study (NCT:04042558) evaluating platinum pemetrexed atezolizumab (+/- bevacizumab) in patients with stage IIIB/IV non-squamous NSCLC with EGFR mutations, ALK rearrangements, or ROS1 fusions who have progressed on targeted therapy. Another randomized phase III multicenter open label study (NCT03991403) was designed to evaluate the efficacy of atezolizumab in combination with carboplatin, paclitaxel, bevacizumab in approximately 228 TKI (tyrosine kinase inhibitor) pretreated patients with stage IV non-squamous non-small cell lung cancer with activating EGFR mutations or ALK translocations, but results are not yet available.

**Table 4:** Concluding remarks clinical studies in ALK mutated advanced non-small cell lung cancer receiving immune checkpoint inhibitors in combination with anti angiogenic inhibitors.

Study	Total No.of Patients	No. of Patients with ALK Rearrangements	Treatment	Endpoint	subgroup		lines of treatment
M. A. Socinski 2018 (112)	800	33(4.13%)	ABCP		ABCP	BCP	First
				m PFS	8.3m	6.8m	
				m OS	19.2m	14.7m	
				ORR	63.50%	48.00%	

These results also led us to further consider whether inhibition of angiogenesis could be to increase the sensitivity of tumor cells to ICIs. Firstly, tumor angiogenesis induces immunosuppression in the body mainly by maintaining an acidic or hypoxic immunosuppressive tumor microenvironment, thereby inhibiting the maturation of dendritic cells and limiting T cell migration. In addition, studies have found that angiogenic factors (e.g., VEGF) also have immunosuppressive functions, which to some extent inhibit the body’s killing of tumor cells (114). Therefore, the co-inhibitory effect of the combination of ICIS and anti-angiogenic agents on ALK rearranged NSCLC needs to be further investigated.

## Recent progress

### ALK neoantigen and ALK vaccine

The concept of vaccines for the treatment of cancer refers to the introduction of tumor associated antigens (TAAs) into the patient, thereby overcoming the immunosuppressive state of the body caused by the tumor and further inducing body humoral immunity and cellular immunity, thus achieving the purpose of inhibiting tumor growth (115). Vaccines have found applications in the prevention and treatment of cancer, such as recombinant human papillomavirus vaccines in melanoma patients and immune circular personalized neoantigen vaccines (116, 117).

In NSCLC, several related vaccines have been approved for clinical use in some countries and regions, such as the Racotumomab and Cimavax epidermal growth factor (Cimavax-EGF) vaccines (115, 118). ALK is a promising neoantigen that can be applied in the development of ALK mutated cancer vaccines because ALK mutations can trigger the body’s T cell response. Two ALK related peptides (SLAMLDLLHV、GVLLWEIFSL) have been shown to be immunogenic against CD8 + T cell epitopes in ALCL patients (119). In addition, these peptides can induce the body to generate cytotoxic T lymphocytes (CTL) specifically against ALK, which have the ability to kill tumor cells of ALK positive neuroblastoma and ALCL. There are preclinical studies in which vaccination with a plasmid carrying a plasmid encoding an ALK related protein in a mouse model of ALK positive lymphoma induced the generation of specific CD8 + CTLs and γ- Interferons, and ultimately effectively suppressed the local progression and distant metastasis of lymphoma. Chemotherapy combined with an ALK DNA vaccine was additionally found to have a synergistic effect on inhibiting tumor growth *in vivo* and improving survival in mice, while the results of the study found that this ALK vaccine could double the survival of mice with ALK positive NSCLC and reduce the number of tumor cells by 60% (120, 121).

In ALK rearranged NSCLC, the aim of ALK directed vaccines is to convert the immunosuppressive TME into an immunoreactive TME, thereby stimulating the migration of TILs to the tumour microenvironment to kill tumour cells (122). The antigenicity of ALK associated proteins has been well established in human lymphomas, where T and B cells have been found to be immunoreactive for ALK associated proteins by examining CD8 + CTLs, CD4 + helper T lymphocytes directed against ALK epitopes, and ALK associated antibodies (119, 123). Further, human ALK expressed proteins are mainly distributed within the central and peripheral nervous system, with ALK associated proteins rarely found within other systems (14). Since ALK related proteins are less exposed in the body during human development, the ALK protein may serve as an ideal tumor antigen for vaccine development. Voena et al. developed a novel ALK vaccine, which was found to activate a mouse specific tumor cytotoxic response and inhibit tumor growth after application in an ALK positive mouse model of NSCLC. The authors went on to find that mice with high PD-L1 expression present had poor vaccine efficacy, but such mice improved vaccine efficacy when combined with immunosuppressive agents (121). Another ALK vaccine developed using a DNA plasmid has also been found to elicit specific antitumor immune responses *in vivo* in mice with ALK positive NSCLC in preclinical trials (124). Using the protect bioinformatics technology combined with animal experiments, it is possible to develop more therapeutic vaccines against ALK positive NSCLC, which also needs further exploration for other types of ALK mutant cancers.

In addition to evaluating the efficacy of the vaccine alone, studies of vaccines combined with ICIs, vaccines combined with chemotherapy, and vaccines combined with ICIs and chemotherapy have been conducted. Results presented at the 2019 American Association for cancer research (AACR) annual meeting showed that the vaccine combination was highly effective in patients with low PD-L1 expression in the tumor and poor response to nivolumab monotherapy. Currently, phase II clinical trials of CIMAvax-EGF vaccine are well underway (NCT02955290). In addition, clinical trials of CIMAvax-EGF with Pembrolizumab (k drug) for first-line treatment in patients with advanced NSCLC with high expression of PD-L1 are also ongoing. In addition, TG4010 (a poxvirus expressing tumor antigen MUCI and IL-2), an oncolytic virus product developed by transgene, is currently under clinical investigation for non-small cell lung cancer in combination with chemotherapeutic agents (125, 126). Meanwhile a phase 2 trial of Bristol Myers Squibb’s nivolumab (O drug) combination is also underway (NCT02823990). Finally, data from an ongoing phase I/II trial of an adenoviral vaccine expressing MAGE-A3 and mg1-magea3 in combination with pembrolizumab treatment (NCT02879760) are not yet available, which may provide novel evidence for a novel ALK vaccine in combination with immunotherapy regimens.

### CAR-T therapy

Chimeric antigen receptor (CAR)-T cells are a class of artificially developed specific therapeutic T cells whose main function is to specifically recognize specific antigens on the surface of tumor cells, thereby clearing tumor cells in the body. Recently, CAR-T therapy has become one of the effective treatments for hematological malignancies. Preliminary data from a study using car-t cell therapy in patients with advanced NSCLC showed a disease control rate of 55% (127). (NCT03525782)Another clinical trial of CAR-T cell therapy in patients with advanced NSCLC was finally forced to terminate due to severe adverse effects in one patient. (NCT03330834)

Recently, studies have found that ALK related proteins on the surface of tumor cells may be potential targets for CAR-T therapy of ALK positive tumors. Walker et al. (128)designed a CAR-T cell specific for ALK positive tumors, which lysed ALK positive neuroblastoma within the co culture medium. However, CAR-T therapy with respect to ALK rearranged cancers requires further analysis, and this class of tumor cell surface should only be partially a target of CAR-T. Further, in most ALK rearranged malignancies, the ALK kinase expressed by the ALK gene is normally located within cancer cells, but the proteins expressed by ALK fusion partners are often located extracellularly. Therefore, CAR-T therapy for ALK positive cancers needs to take into account not only the ALK kinase, but also the extracellular structures expressed by more important ALK fusion partners such as EML4 or NPM, among others.

### Adoptive T cell therapy

Adoptive cell therapy (ACT), a type of antitumor effect by injecting ex vivo expanded T lymphocytes into patients to improve the body, similar to CAR-T therapy, may become one of the options for patients with solid tumors in the future (129). NSCLC is characterized by a high clonal mutational load, a characteristic that has provided a strong rationale to support the intensive study of clonal neoantigen reactive T cells (cNeT) (129). The first application of this therapeutic modality in the clinic was ATL001, a personalized cNeT product obtained by applying the PELEUS bioinformatics platform (developed utilizing TRACERx research data in the United Kingdom) to matched carcass and blood samples from patients with NSCLC as part of a tissue collection study (NCT03517917) (130). The CHIRON study is the first ongoing open label multicenter phase I/IIA study investigating the safety and efficacy profile of ALT001 in patients with advanced NSCLC (NCT04032847).

Since the first report of this therapy in 1986 (131), tumor infiltrating lymphocytes (TILs) have been isolated in a variety of solid tumors and have given satisfactory results in clinical trials (132–139). In 2019, Sarnaik et al., on data from cryopreserved autologous TILs (ln-144, lifileucel) for the treatment of 66 patients with advanced metastatic melanoma, found a disease response rate of 38% and a disease control rate of 80% (129). A recent single arm open label phase 1 trial (NCT03215810) of TILs in 20 patients with advanced NSCLC (two with EML4-ALK translocation mutations) after immunosuppressant resistance. Of the 13 evaluable patients, three had a confirmed response and 11 had a reduction in tumour burden, with a mean best change rate of 35%. The simultaneous discovery that autologous TILs for cell therapy are generally safe and clinically active in a subset of patients with advanced NSCLC might represent a novel therapeutic strategy for ALK mutated advanced lung cancer (140).

Current studies of tumor infiltrating lymphocyte (TIL) therapy for NSCLC are limited, but the high mutational load of NSCLC itself and the high responsiveness to ICIS suggest that this class of cancer is a predictable treatment option for ACT. There was clinical experience in the 1990s with the use of TILs in the treatment of NSCLC (141). In this study, TIL cultures were successfully amplified from tumors surgically removed from patients with stage II and III lung cancer. It was found that the 3-year OS of patients with stage III disease was significantly improved, and the local recurrence rate was also significantly lower, providing preliminary evidence that TIL therapy can improve the prognosis of patients with advanced NSCLC.

Recent intensive studies in lung cancer found the feasibility of cNeT as an emerging therapy for NSCLC (142). Because clonal neoantigens are rarely present within normal tissues but may be present on all tumor cells. In addition, because TILs are not generated by transgenic technology, the immune tolerance of patients to clonal reactive T cells is theoretically better. With further research on the therapeutic approach of cNeTs, it is expected in the future to provide better treatment options for patients with ALK rearranged NSCLC who develop resistance to ALK TKIs.

### Anti ALK antibody

Antagonistic or neutralizing antibodies are one of the effective therapeutic means against malignant tumors. Such as cetuximab (anti-EGFR) in head and neck cancer and trastuzumab (anti-HER2) in breast cancer, among others. Since the ALK receptor is mainly distributed on the cell surface, neutralizing antibodies can be used to treat ALK mutated related tumors. In addition, the expression of ALK related genes is mainly distributed in the nervous system, making anti ALK antibodies less harmful to normal tissues. Recently, research targeting anti ALK antibodies is constantly being explored, and some newly developed monoclonal antibodies against ALK have been confirmed to inhibit the growth of ALK positive tumor cells in *in vitro* experiments (143).

Injection of a human single chain variable fragment (scFv) antibody against the ALK ligand-binding domain (LBD) in a mouse model has been studied, and ALK positive glioblastoma growth was found to be inhibited. Stylianou et al (144), demonstrated significant shrinkage of glioblastoma tumors expressing an anti ALK scFv by tetracycline compared to tumors not expressing the antibody, providing a rationale for the clinical use of anti ALK scFv in the treatment of ALK positive tumors. In addition, anti ALK antibodies have also been found to specifically inhibit neuroblastoma growth in ALK mutated human neuroblastoma derived cell lines (145). An additional study found that ALK antibodies were detected in the serum of 13 of 21 patients with ALK translocated NSCLC and none of the patients who were ALK negative (26). This suggests that for ALK positive NSCLC patients, enhancing the pre-existing anti ALK immune response may derive additional benefit.

## Conclusion

ALK rearrangements are present in 5-6% of patients with NSCLC and confer high sensitivity to ALK TKIs. After ALK rearranged NSCLC patients become resistant to one or more ALK TKIs, the next treatment options include chemotherapy, immunotherapy, and a combination of chemotherapy and immunotherapy, among others. Many preclinical studies have found higher PD-L1 expression on tumor cells in patients with ALK rearranged NSCLC than on other types of lung cancer cells, but clinical trials of single agent immunotherapy have not yielded significant clinical benefit. There are studies that consider ALK rearranged NSCLC driving the organism to generate an immunosuppressive TME and thus cause tolerance to ICIs in such patients. To further explore the efficacy of immunotherapy in patients with ALK rearranged NSCLC, data from clinical trials combining immune checkpoints with treatment strategies such as ALK TKIs, chemotherapy and anti angiogenic agents are summarized. Most treatment regimens targeting immunotherapy in combination with ALK TKIs suffer from significant toxicities and low efficacy. The simultaneous discovery of potential toxic effects of certain tyrosine kinase inhibitors in patients with ALK rearrangement who had received previous immunotherapy provides evidence for the need for further refinement of genetic testing before treatment of patients with lung cancer. No significant clinical benefit was also found in the combination of immunotherapy combined with chemotherapy, but the combination of immunotherapy with antiangiogenic regimens may provide further prolongation of survival for patients after ALK TKI resistance.

ALK directed vaccines convert an immunosuppressive TME into a highly reactive TME, and their significant efficacy has been demonstrated in preclinical studies, together with the discovery of synergistic antitumor effects of the vaccine in combination with chemotherapy or immunotherapy. Studies of combination regimens of vaccines still require validation in a large number of preclinical and clinical studies, and the case for sustained efficacy based on vaccine therapy requires further exploration. The efficacy of CAR-T cell therapy mainly depends on the specific recognition of tumor cell surface antigens and possible damage to surrounding tissues. ACT therapy as a kind of CAR-T cell therapy, infusing ex vivo expanded T cells into patients improves the body’s antitumor ability. The requirement of ACT therapy for the hypermutability of the tumor cells themselves and for hyperresponsiveness to immunotherapy, indicates the potential significance of ALK rearranged NSCLC to this therapy. In an attempt to further improve the survival prognosis of patients with ALK rearranged NSCLC lung cancer, monoclonal antibodies against ALK related proteins are in a pipeline and have demonstrated certain efficacy in preclinical studies.

## Data availability statement

The original contributions presented in the study are included in the article/supplementary material. Further inquiries can be directed to the corresponding author.

## Author contributions

SSH provided the initial idea for this review. RBQ and YYY were in charge of data acquisition and drafting of the article. MS revised the article. All authors contributed to the article and approved the submitted version.
